# Spontaneous Activity Patterns Are Altered in the Developing Visual Cortex of the *Fmr1* Knockout Mouse

**DOI:** 10.3389/fncir.2019.00057

**Published:** 2019-09-26

**Authors:** Juliette E. Cheyne, Nawal Zabouri, David Baddeley, Christian Lohmann

**Affiliations:** ^1^Department of Synapse and Network Development, Netherlands Institute for Neuroscience, Amsterdam, Netherlands; ^2^Auckland Bioengineering Institute, University of Auckland, Auckland, New Zealand; ^3^Department of Functional Genomics, Center for Neurogenomics and Cognitive Research, VU University Amsterdam, Amsterdam, Netherlands

**Keywords:** fragile X mental retardation, *in vivo* calcium imaging, sensory integration, 2-photon microscopy, transgenic mouse

## Abstract

Fragile X syndrome (FXS) is the most prevalent inherited cause of autism and is accompanied by behavioral and sensory deficits. Errors in the wiring of the brain during early development likely contribute to these deficits, but the underlying mechanisms are unclear. Spontaneous activity patterns, which are required for fine-tuning neuronal networks before the senses become active, are perturbed in rodent models of FXS. Here, we investigated spontaneous network activity patterns in the developing visual cortex of the *Fmr1* knockout mouse using *in vivo* calcium imaging during the second postnatal week, before eye opening. We found that while the frequency, mean amplitude and duration of spontaneous network events were unchanged in the knockout mouse, pair-wise correlations between neurons were increased compared to wild type littermate controls. Further analysis revealed that interneuronal correlations were not generally increased, rather that low-synchronization events occurred relatively less frequently than high-synchronization events. Low-, but not high-, synchronization events have been associated with retinal inputs previously. Since we found that spontaneous retinal waves were normal in the knockout, our results suggest that peripherally driven activity is underrepresented in the *Fmr1* KO visual cortex. Therefore, we propose that central gating of retinal inputs may be affected in FXS and that peripherally and centrally driven activity patterns are already unbalanced before eye opening in this disorder.

## Introduction

Autism spectrum disorders (ASD) are a group of neurodevelopmental disorders that have been characterized traditionally by core features such as weak social communication, restricted interests, and repetitive behaviors. More recently, it has become clear that these disorders are also associated with compromised sensory processing, including vision (Kogan et al., 2004; Knoth et al., 2014; Dickinson et al., 2016; Ethridge et al., 2017; Yamasaki et al., 2017). Changes in sensory perception may underlie the complex behavioral traits described previously (Baum et al., 2015; Yamasaki et al., 2017; Rais et al., 2018). Studies in animal models of ASD found alterations in sensory perception and plasticity as well (Dolen et al., 2007; Berzhanskaya et al., 2016; Orefice et al., 2016; He et al., 2017; Goel et al., 2018; Wen et al., 2019). Miswiring of central sensory pathways may underlie these symptoms (Yamasaki et al., 2017; Goswami et al., 2019); however, the developmental mechanisms that cause miswiring of sensory pathways in ASD and neurodevelopmental disorders in general are unknown (Di Martino et al., 2014; Park et al., 2016).

The wiring of neuronal networks is driven by molecular cues early on and later refined by activity-dependent mechanisms (Sanes and Yamagata, 2009; Kirkby et al., 2013). Before the onset of sensation, spontaneous activity is required for fine-tuning synaptic connections for sensory processing; thereafter, experience further adapts sensory networks to the prevalent conditions in the environment (Villers-Sidani et al., 2007; Caras and Sanes, 2015; Soutar et al., 2016). Perturbed mechanisms at all these developmental stages may contribute to neurodevelopmental syndromes (Meredith, 2015; Sanders, 2015).

Fragile X syndrome (FXS) is the most common genetic cause of autism and has been investigated extensively in patients and animal models (Hagerman et al., 2017), in particular the *Fmr1* knockout mouse (The Dutch-Belgian Fragile X Consortium, 1994; Mientjes et al., 2006). These studies confirmed sensory phenotypes in both humans and mice (Kogan et al., 2004; Dolen et al., 2007; Knoth et al., 2014; Zhang et al., 2014) and these phenotypes are most likely caused by compromised synaptic connectivity (Bureau et al., 2008; Gibson et al., 2008; La Fata et al., 2014). Importantly, spontaneous activity patterns are perturbed during development, suggesting that errors in early activity-dependent synaptic refinement may impair synaptic connections in the FXS brain. For example, spontaneous network events in the somatosensory cortex of the *Fmr1* KO mouse show increased correlations (Goncalves et al., 2013), which may render these activity patterns less suitable for refining developing networks (Leighton and Lohmann, 2016).

Here, we investigated spontaneous activity patterns in the developing primary visual cortex of the *Fmr1* KO mouse (Mientjes et al., 2006) before eye opening. We find increased inter-neuronal correlations in the developing visual cortex, similar to previous findings in the somatosensory cortex (Goncalves et al., 2013). Further analysis suggested, however, that there is not a general increase in correlations, but rather a relative decrease of low- vs. high-synchronicity network events. Low-synchronicity events (L-events) have previously been associated with inputs from the retina (Siegel et al., 2012). Thus, our results suggest that retinally driven activity is underrepresented in cortical *Fmr1* KO activity patterns. Since we show here that retinal waves are normal in the knockout, we propose that central gating in the ascending visual pathway may be affected in FXS, even before the onset of vision.

## Materials and Methods

### Animals

All experimental procedures were approved by the Institutional Animal Care and Use Committee of the Royal Netherlands Academy of Arts and Sciences. The *Fmr1* KO mouse line used here was previously backcrossed to C57Bl/6J mice at least seven generations (Mientjes et al., 2006; de Vrij et al., 2008). The mice used in this study were bred from heterozygote *Fmr1* (*Fmr1*^+/–^) mothers and wild type fathers (WT, C57Bl/6J). Only male KOs (*Fmr1*^*y/*–^) were used in experiments, with littermate (wild type, *Fmr1*^*y/+*^) males as controls. Experiments and initial analysis were performed blind to the genotype. There were no significant differences in weight (control: 6.79 ± 0.32 g, *n* = 20; FX: 6.51 ± 0.27 g, *n* = 18; *p* > 0.05) or age (control: 10.20 ± 0.29 days, *n* = 20, FX: 10.39 ± 0.26 days, *n* = 18, *p* > 0.05) between KOs and WTs for the cortical experiments. In the retinal experiments the weight (control: 5.58 ± 0.31 g, *n* = 15, FX: 5.34 ± 0.22 g, *n* = 17, *p* > 0.05) and age (control: 9.60 ± 0.31 days, *n* = 15, FX: 9.94 ± 0.20 days, *n* = 17, *p* > 0.05) also did not differ between KOs and WTs. All of the mice had closed eyes at the time of the experiment which fits with previous research showing that C57Bl/6J mice open their eyes at P12–P14 (Rochefort et al., 2009).

### Genotyping

Mouse genotypes were determined *post hoc* by polymerase chain reaction (PCR) using the following primers: for KOs (GCCTCACATCCTAGCCCTCTAC and CCCACTGG GAGAGGATTATTTGGG) and for WTs (GCCTCACATCC TAGCCCTCTAC and CCCACAAAGTTGATTCCCCAGA). Tail samples were digested overnight with proteinase K (0.2 μg/μL) in 500 μL tail lysis buffer (in mM: 100 Tris–HCl, 5 EDTA, 200 NaCl, and 0.2% SDS) at 56°C. Proteins were pelleted by centrifugation (14,000 rpm for 3 min); DNA was isolated and precipitated with 500 μL of isopropanol. Following centrifugation (14,000 rpm for 1 min) the DNA pellet was dried and subsequently resuspended in 50 μL Tris–EDTA buffer. The PCR mix was prepared by adding: 2.6 μL of 10× buffer, 0.25 μL dNTPs (20 mM), 0.5 μL primer mix (10 μM), 0.15 μL Taq polymerase, and milliQ water up to a total of 25 μL for each sample. 1 μL of mouse DNA was added and the mixture was kept at 95°C for 5 min followed by 40 cycles (10 s at 95°C, 20 s at 60°C, and 45 s at 72°C) and 72°C for 10 min to finish.

### Visual Cortex Imaging

*In vivo* imaging experiments were performed as described previously (Siegel et al., 2012). Briefly, P8–14 mice were anesthetized with isoflurane (2% in 1.7 L/min O_2_), attached to a head bar with super glue and stabilized with dental cement (Heraeus Kulzer). Isoflurane was then reduced to 0.7% for the remainder of the experiment during which temperature was maintained at 36–37°C and heart beat was continuously monitored (control: 329.15 ± 16.17 bpm, *n* = 20, FX: 323.89 ± 17.94 bpm, *n* = 18, *p* > 0.05). At this level of isoflurane, animals remained anesthetized as they showed limited movement and a lack of reflexes. However, their breathing was rapid and shallow, and heart rates were high indicating that they were lightly anesthetized, as they lacked low gasping respiration and low heart rates typical in deeply anesthetized mice of this age. A craniotomy was made above the visual cortex without perforating the dura. The exposed cortical surface was kept moist with cortex buffer (in mM: 125 NaCl, 5 KCl, 10 glucose, 10 HEPES, 2 MgSO_4_ and 2 CaCl_2_, pH 7.4). A large population of layer 2/3 neurons was then labeled by bolus loading with the calcium-sensitive dye Oregon Green BAPTA-1 AM (OGB1-AM, Life Technologies, O-6807) or Cal-590 AM (AAT Bioquest, 20510) diluted in 4 μL pluronic F-127 (20% solution in DMSO, Life Technologies, P-3000MP), and 36 μL dye buffer (in mM: 150 NaCl, 2.5 KCl, and 10 HEPES, pH 7.4). Dye was injected into layer 2/3 through a glass pipette (3–7 MΩ) using a picospritzer (12 min, 10–12 psi, Toohey). After 1 h the craniotomy was covered with 1.5% agarose for extra stability and imaging was performed to monitor spontaneous network activity in the same set of neurons (Mean cell number: Control: 91.80 ± 10.03, *n* = 20, FX: 80.17 ± 6.84, *n* = 18, *p* > 0.05) for approximately 40 min (Mean imaging time: Control: 38.31 ± 2.69 min, *n* = 20, FX: 43.42 ± 2.78 min, *n* = 18. Total duration recorded: Control: 766 min, FX: 781 min). Consecutive xyt-stacks (256 × 256, 600 nm pixel size, 5 Hz) were obtained through a 40× water-immersion objective (0.8 NA, Olympus) with a two-photon microscope (Movable Objective Microscope, Sutter Instrument or A1R-MP, Nikon) and a mode-locked Ti:Sapphire laser (at λ = 810 nm; Spectra Physics or Coherent) controlled by custom made Labview (National Instruments) software or ScanImage (Pologruto et al., 2003). 5-min recordings were obtained with only short breaks for re-focusing when necessary.

### Visual Cortex Image Analysis

Recordings of spontaneous network activity in the cortex were analyzed with ImageJ (NIH) and custom-written Matlab scripts (MathWorks). To remove movement artifacts and align all recordings we performed image alignment based on the enhanced correlation coefficient algorithm (Evangelidis and Psarakis, 2008). ΔF/F_0_ stacks were generated by subtracting and dividing each frame by the baseline fluorescence (F_0_). Regions of interest (ROIs) were placed on cells that showed clear activity and were visible in all recordings. Glial cells in the field of view showed elevated basal intensity and were not active. All included ROIs were neuronal. ΔF/F_0_ traces were obtained by calculating the mean intensity within the ROI for each frame. Increases in fluorescence intensity, which reflect increases in the intracellular calcium concentration due to action potential firing, were then detected semi-automatically for all ROIs and the maximum amplitudes and timings were determined. The detection threshold was adjusted for each experiment (at least 2× the noise) but remained the same within an experiment. A network event was defined as activity across multiple neurons during consecutive frames (<15 frames separation). The participation rate for each event was determined by summing the number of active cells and dividing by the total number of ROIs. Events with less than 20% participation were not analyzed further. Previously we found that two types of activity occur in the visual cortex at this developmental age: L-events are low participation (20–80%) and low amplitude events that are generated in the retina and transmitted to the cortex, while H-events are high participation (>80%) and high amplitude events that are generated within the cortex (Siegel et al., 2012). Here we also divided the data into H-events and L-events based on participation (above or below 80%). We found that varying the cut-off between 70 and 90% did not affect the results presented in this study (not shown).

To analyze synchrony, we calculated Pearson’s correlations for each neuron pair in each experiment. Correlations were calculated on binarized activity traces of each neuron where each neuron was given a value of 0 for each inactive frame and a value of 1 for each frame of each burst it was active in. Burst duration was determined as the difference between the peak frame of the first and last cell to become active. To compare the correlation means we averaged across all pairs within 200 μm for each animal. The distance between the two cells was calculated as a straight line connecting the center of each ROI. We excluded animals that had fewer than 10 events in the recorded time (6 mice were excluded, 4 WTs and 2 KOs).

### Retinal Imaging

Postnatal day 8–11 (P8–11) mice were anesthetized by inhalation of isoflurane (2% in 1.7 L/min O_2_) and killed by decapitation. The eyes were removed and placed into ice cold modified Hank’s balanced salt solution (HBSS, Life Technologies, in mM: 3.26 CaCl_2_, 0.493 MgCl_2_, 0.406 MgSO_4_, 5.33 KCl, 0.441 KH_2_PO_4_, 4.17 NaHCO_3_, 138 NaCl, 0.336 Na_2_HPO_4_, and 5.56 D-glucose) and dissected to isolate the retinas. Ganglion cells were labeled by injection of OGB1-AM (prepared as for visual cortex imaging) just below the inner limiting membrane with a glass pipette (1.5–5 MΩ, 3 min, 15 psi). After 1 h cells retinas were placed in a heated chamber (35°C) and imaged to monitor spontaneous network activity for ∼ 45 min (Mean imaging time: Control: 47.33 ± 1.18 min, *n* = 15, FX: 41.76 ± 1.05 min, *n* = 17. Total duration recorded: Control: 710 min, FX: 710 min). Consecutive xyt-stacks (500 × 500 pixels, 1.6 μm pixel size, 10 Hz) were obtained through a 20× water-immersion objective (0.5 NA, Olympus) with a CCD camera (iXon+; Andor Technology) and LED-based excitation illumination (pE-2; CoolLed) controlled by custom-built software (Labview, National Instruments).

### Retinal Image Analysis

Recordings of spontaneous network activity in the retina were analyzed in a similar way to the cortical recordings. However, due to the large field of view and resulting small size of neuronal somas we did not analyze retinal activity in individual neurons. Instead, we downsized the images into 10 × 10 pixel bins and analyzed activity in the resulting 100 pixels. ΔF/F_0_ stacks were generated as described above and the ΔF/F_0_ traces were obtained for each pixel. Activity was then detected as for the cortical experiments except the same threshold was used for all experiments.

Retinal wave front velocities were quantified using custom python code implemented as part of the python-microscopy project^1^. Full resolution ΔF/F_0_ stacks were smoothed with a Gaussian (radius of 10 pixels) to reduce noise. The positions of wave front peaks in each frame were estimated as the zero-crossings of the time derivative of intensity as follows: (1) A low threshold was used to establish a mask of areas where calcium was elevated and to eliminate the inactive areas whose intensity was roughly constant over time, (2) in pixels above this threshold, an approximate temporal derivative of the intensity (Δ_*t*_*I*) was calculated by taking the difference between consecutive frames, (3) zero-crossings were detected by finding all pixels where |Δ_*t*_*I*| was less than a threshold (chosen to give a gap-free wave front ∼4–5 pixels wide) and skeletonizing the resulting masks. The direction of propagation of these wave fronts was estimated using a regularized version of optical flow algorithm described previously (Fleet and Weiss, 2006) applied to the filtered intensity data. Velocities were then estimated at each point on the wave front by looking along the optical flow direction, extracting the closest position of the wave front along this vector in each of the 5 frames before and after the current frame, and performing a linear least-squares fit to these positions as a function of time.

### Statistics

To test for statistical differences between groups we used the Wilcoxon rank sum test. Data are presented as means ± SEM with n as the number of animals.

## Results

**FIGURE 1:**
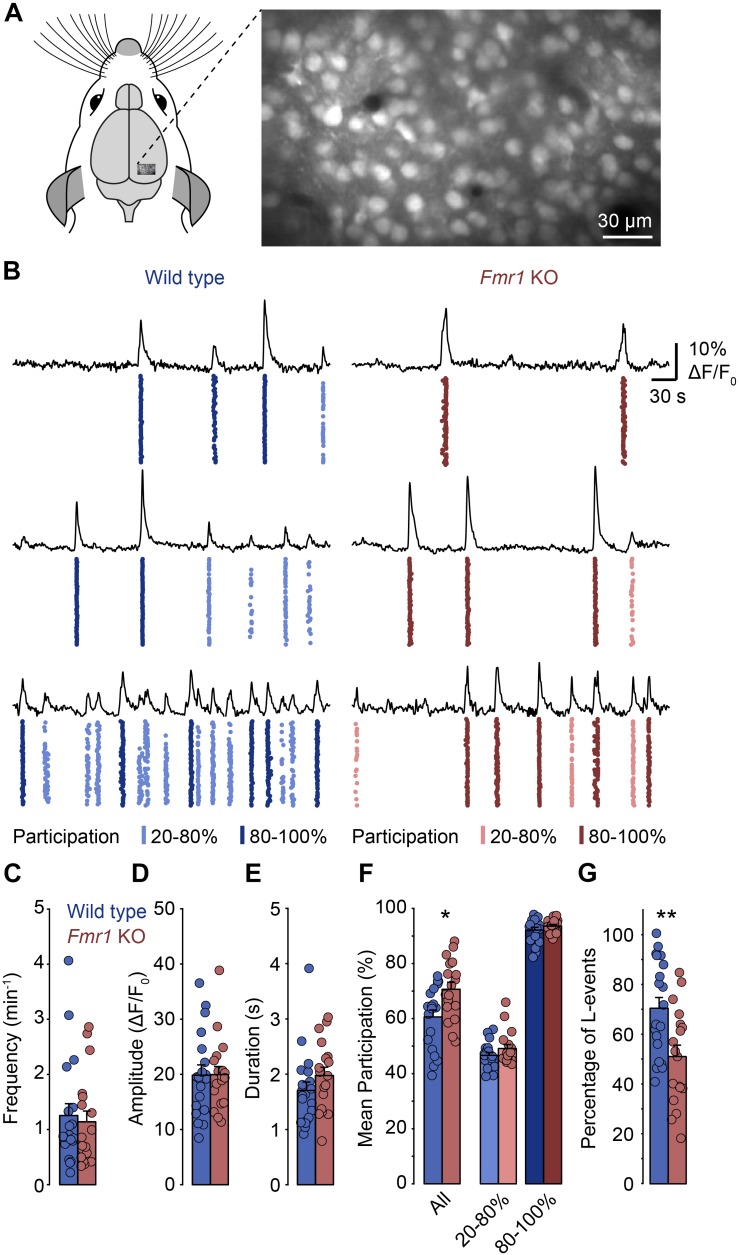
In *Fmr1* KO mice, spontaneous network events in layer 2/3 of the primary visual cortex show increased neuronal participation. **(A)** Experimental approach for network imaging in the developing mouse primary visual cortex (P8–P14). Layer 2/3 neurons were loaded with a calcium indicator (OGB-1-AM) and imaged for approximately 40 min using two-photon microscopy to measure neuronal network activity. **(B)** Example recordings of layer 2/3 neuronal activity in wild type and *Fmr1* KO mice (right). For each panel the top trace shows the mean activity of all imaged cells in the recording. The plot below shows the activity of all neurons in the field of view with each dot representing an activation of a single neuron. The event participation is indicated by the color, for L-events (20–80% participation, lighter), and H-events (80–100% participation, darker). The examples reflect the different levels of spontaneous activity we observed in individual animals. **(C–E)** Frequency, amplitude and duration of network events were unchanged in *Fmr1* KO mice. **(F)** Neuronal participation was increased in *Fmr1* KOs (^∗^*p* = 0.011, Wilcoxon rank sum test), but within L- and H-events (20–80 and >80% respectively), participation was indistinguishable between both groups. **(G)** The percentage of L-events was decreased in *Fmr1* KO mice (^∗∗^*p* = 0.007, Wilcoxon rank sum test).

To determine whether spontaneous network activity patterns are altered in the developing visual cortex of FXS mice we performed *in vivo* calcium imaging in the primary visual cortex of lightly anesthetized *Fmr1* knockout mice (*Fmr1*^–^*^/y^*) and wild type littermate controls (*Fmr1*^+/y^) at postnatal day (P) 8–14 (Figure 1). Previously, we found that low levels of anesthesia (0.7 – 1% isoflurane) reduce the frequency of spontaneous network events, but do not change their basic properties (Siegel et al., 2012). We labeled layer 2/3 neurons with a fluorescent calcium indicator (Oregon Green BAPTA-1 or Cal-590) by bolus injection (Figure 1A) and recorded neuronal activity using two-photon imaging. Increases in somatic calcium, which reflect action potential firing, were monitored continuously in large populations of neurons for approximately 40 min (Figure 1B).

In both wild type and *Fmr1* KO mice, we observed repetitive events of synchronized network activity as described previously (Figure 1B; Hanganu et al., 2006; Golshani et al., 2009; Rochefort et al., 2009; Colonnese et al., 2010; Siegel et al., 2012). In most animals, there was no or little activity in between these network events, as is typically observed during this developmental stage. We found that the frequency, the mean amplitude and the duration of network events were indistinguishable between *Fmr1* KOs and controls (Figures 1C–E). In contrast, the mean participation was significantly higher in *Fmr1* KOs than in wild type animals (Figure 1F). Previously, we had identified two activity patterns in the developing visual cortex before eye-opening: L-events with low participation rates that are dependent on retinal wave activity and H-events where almost all neurons participate, which are independent of retinal inputs (Siegel et al., 2012). When we separated network events in the present data sets and compared participation rates within L-events (20–80% participation) and H-events (80–100% participation), we found that within these groups there was no change in participation (Figure 1F). This suggested that in *Fmr1* KO mice overall participation was not generally increased, but that the relative contribution of L- and H-events differed in the knockout mouse. Indeed, we found that the proportion of L-events was decreased in the *Fmr1* KO mouse (Figure 1G).

**FIGURE 2:**
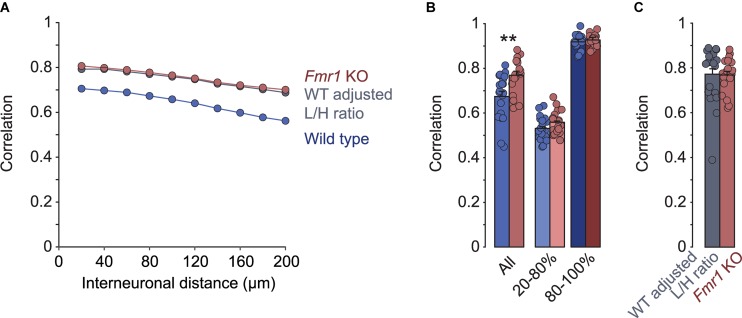
Differences in correlations of network activity between *Fmr1* KO mice and wild type littermates can be explained by changes in L- and H-type activity patterns. **(A)** Mean pairwise correlations binned for inter-neuronal distance in *Fmr1* KOs, wild types, and wild types adjusted for L/H-event ratios. In *Fmr1* KOs correlations were increased across all distance bins. Randomly deleting L-events from wild type activity patterns to mimic the reduced L/H-event ratio in KOs compensated the difference fully. **(B)** Correlations differed significantly when all events were compared (^∗∗^*p* = 0.002, Wilcoxon rank sum test), but were indistinguishable between *Fmr1* KO and wild type when activity events were separated into L- and H-events. **(C)** When matching the L/H-event ratios between *Fmr1* KOs and wild type animals, correlations became virtually identical (*Fmr1* KO data is from **B**, “All”, for comparison).

A previous study found that in the somatosensory cortex of *Fmr1* KO mice, neuronal activity was more correlated than in wild type controls (Goncalves et al., 2013). To compare these results with the primary visual cortex, we analyzed pair-wise correlations of the calcium traces of layer 2/3 neurons in our recordings. Like in the somatosensory cortex, we observed a decrease in correlations with increasing distance between them, in both *Fmr1* KO (*R*^2^ = 0.99, *p* < 0.001) and control (*R*^2^ = 0.98, *p* < 0.001) mice (Figure 2A). In addition, correlations were higher in the *Fmr1* KO mouse compared to controls across all distance bins (Figures 2A,B). We did not observe significant age related changes of these correlations or their differences between genotypes (Supplementary Figure 1). Surprisingly, we found that correlation coefficients when determined for L- and H-events separately were virtually identical in *Fmr1* KO and control mice (Figure 2B). Again, this observation suggested that we did not observe a global increase in correlations, but rather a shift toward a higher fraction of high-correlation network events (H-events). To test the plausibility of this idea we randomly deleted L-events from the control dataset to match the percentage of L-events of *Fmr1* KO and control recordings (WT adjusted L/H ratio). This adjustment increased the overall correlations to the same level as those seen in *Fmr1* KO mice (Figures 2A,C), confirming that a shift in L-/H-event ratio could explain the increased correlations in the *Fmr1* KO visual cortex.

**FIGURE 3:**
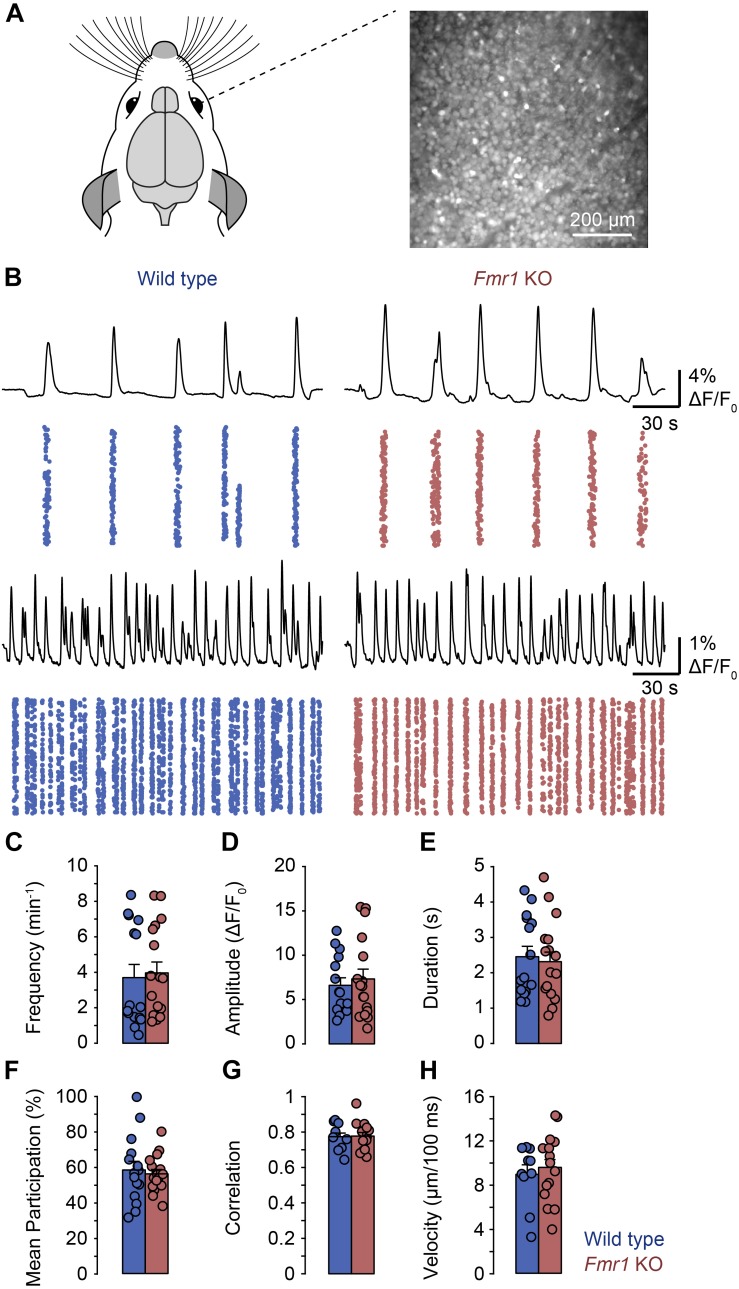
General properties of retinal waves were not different in *Fmr1* KO mice and littermate controls in whole mount recordings. **(A)** Experimental approach for imaging whole-mount mouse retinas (P8–P11). Retinal ganglion cells were loaded with OGB-1-AM and imaged for approximately 40 min using wide-field microscopy. **(B)** Example recordings of retinal waves in wild type (left) and *Fmr1* KO mice (right). For each panel the top trace shows the mean activity of the field of view. The plot below shows the activity of all regions of interest (ROIs) in each field of view with each dot representing an activation in a single ROI. ROIs were generated by rasterizing each image into 10 × 10 pixel bins. The examples reflect the different frequencies of retinal waves we observed in individual retinas. **(C–H)** Frequency, amplitude, duration, participation, correlation, and velocity of retinal waves were unchanged in *Fmr1* KO mice.

Since we previously found that L-, but not H-events are dependent on retinal wave activity, the present observations suggested that the representation of inputs from the sensory periphery is reduced in comparison to intrinsically generated activity patterns. Therefore, we asked whether retinal waves were affected in the *Fmr1* KO mouse. We performed calcium imaging in retinal whole mounts from P8–P11 *Fmr1* KO and control mice (Figure 3A). We observed retinal waves in both preparations (Figure 3B) and found no differences in frequency, amplitude, duration, participation or correlation between them (Figures 3C–G). In addition, we analyzed the velocity of retinal waves but found no difference between *Fmr1* KO and control mice (Figure 3H). Thus, peripheral activity is underrepresented in the *Fmr1* KO visual cortex, despite normal retinal waves, suggesting differences in central processing of peripheral activity in FXS.

## Discussion

Neurodevelopmental disorders are associated with aberrant spontaneous activity patterns during early stages of brain development. Here, we show that in the visual cortex of the *Fmr1* KO mouse, neuronal correlations are increased compared to wild type littermates. The distribution of correlations suggest that this is not a consequence of generally increased correlations, but rather a redistribution of the contribution of two distinct, previously described activity patterns. Specifically, we see a relative decrease in those activity patterns that are dependent on retinal inputs even though retinal waves are unchanged in this FXS mouse model.

Like in the somatosensory cortex (Goncalves et al., 2013), we observe here an increase in the mean correlation coefficient when we compare activity patterns between pairs of neurons in *Fmr1* KO mice and wild type littermates. This increase is quantitatively and qualitatively very similar to the increase in the somatosensory cortex, as it affects pairs of neurons of all distances and translates to a change of approximately 10–20%. While in the somatosensory cortex differences become most pronounced by P14, we see differences in the visual cortex already at P8–P14. These differences are apparent in lightly anesthetized animals, whereas a significant difference between KO and wild type has been described in the somatosensory cortex only in awake animals (Goncalves et al., 2013). Despite the differences in detail between the primary visual and somatosensory cortices, these studies show that cortical activity patterns in *Fmr1* KO mice are associated with increased neuronal correlations. Similar findings have been observed at P0–P7 (La Fata et al., 2014).

Surprisingly, we find that increases in correlations do not distribute evenly across all types of events. When splitting events into high and low participation activity as described previously (Siegel et al., 2012) participation rates and correlations are unchanged in either group. We conclude therefore, that correlations are not generally increased, but rather that a change in contribution of these two types of activity patterns causes the increased mean correlations in the *Fmr1* KO visual cortex. This conclusion is supported by (1) the overall reduction in the L-/H-event ratio and (2) the fact that matching the L/H-event ratio between *Fmr1* KO and control activity fully equalizes the correlations between both conditions. Since L-events, but not H-events, have been associated with retinal inputs (Siegel et al., 2012), these observations suggest that peripheral activity is underrepresented in the *Fmr1* KO visual cortex before eye opening. Peripheral and central inputs may have complementary functions in cortical wiring (Siegel et al., 2012; Leighton and Lohmann, 2016), consequently, changes in their relative frequencies are likely to perturb synapse development.

Deficits in photoreceptor function have been described in the *Fmr1* KO mouse (Rossignol et al., 2014) therefore, we tested whether changes in early retinal activity could explain this difference. However, retinal waves are not affected in the *Fmr1* KO mouse, since their frequency, amplitude, and correlation are indistinguishable between KOs and controls.

What could explain the reduced representation of retinal inputs in the *Fmr1* KO visual cortex? A good candidate might be alterations in inhibitory neuron function. Early differences in inhibitory function have been implicated in neurodevelopmental disorders in general and FXS in particular (Marin, 2012; Le Magueresse and Monyer, 2013; Cellot and Cherubini, 2014; Tyzio et al., 2014; Goel et al., 2018). Furthermore, different types of interneurons are at pivotal positions within cortical networks to gate specifically bottom-up or horizontal activity. While PV neurons selectively target bottom-up connections, other interneurons, like the somatostatin expressing interneuron type, regulate horizontal activity spread (Tremblay et al., 2016; van Versendaal and Levelt, 2016; Wood et al., 2017). In addition, during development, cortical interneurons control spontaneous and early sensory evoked activity in a cell-type specific manner (Marques-Smith et al., 2016; Tuncdemir et al., 2016; Che et al., 2018). Therefore, selective alterations e.g., in PV neuron function could specifically decrease the effectiveness of ascending sensory pathways and thereby dampen cortical inputs from the periphery (Khazipov et al., 2013; Goel et al., 2018). Alternatively or in addition, differences in the excitability or synaptic function of excitatory connections at the level of the cortex or the lateral geniculate nucleus (Murata and Colonnese, 2016) might be involved as well. For example, a delay in the maturation of thalamo-cortical synapses has been described in the somatosensory cortex of *Fmr1* KO mice (Harlow et al., 2010). A similar delay of synapse development in the ascending visual pathways could explain the reduced transmission of retinal activity into the cortex during the second postnatal week, too.

Imbalanced contribution of external vs. internal activity streams have been observed in neurodevelopmental disorders (Courchesne and Pierce, 2005; Geschwind and Levitt, 2007). Thus, the findings described here, suggest that network imbalances already manifest early in development, before the onset of sensation. If this can be generalized beyond FXS, diagnosis of neurodevelopmental disorders may be possible earlier than previously thought and thus facilitate earlier and more promising treatments in the future.

## Data Availability

The datasets generated for this study are available on request to the corresponding author.

## Ethics Statement

The animal study was reviewed and approved by the Animal Care and Use Committee of the Royal Netherlands Academy of Arts and Sciences.

## Author Contributions

JC and CL conceived and designed the study, and wrote the manuscript. JC and NZ collected the data. JC and DB contributed to the data and analysis of the tools. JC performed the analysis, and contributed to all except the retinal velocity analysis, which was written by DB.

## Conflict of Interest Statement

The authors declare that the research was conducted in the absence of any commercial or financial relationships that could be construed as a potential conflict of interest.

## Footnotes

1 www.python-microscopy.org
