# Supplementary figures and images for: Spontaneous Activity Patterns Are Altered in the Developing Visual Cortex of the Fmr1 Knockout Mouse
Source: Front Neural Circuits. 2019 Sep 26;13:57. doi: 10.3389/fncir.2019.00057 (PMC6775252; doi:10.3389/fncir.2019.00057)

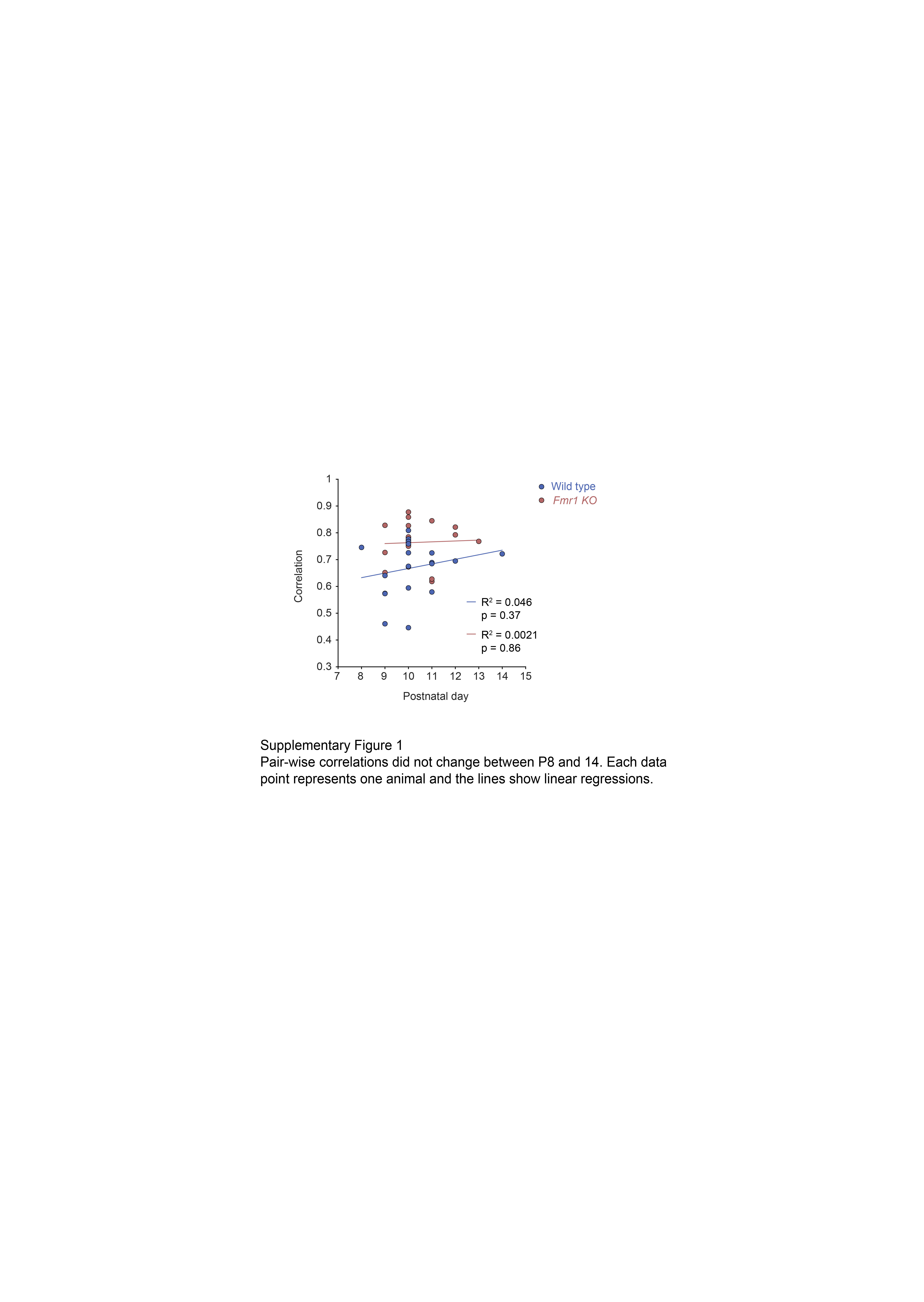

Supplement: Supplementary file 1 [file Image_1.JPEG]
